# Gravity sensing, a largely misunderstood trigger of plant orientated growth

**DOI:** 10.3389/fpls.2014.00610

**Published:** 2014-11-05

**Authors:** David Lopez, Kévin Tocquard, Jean-Stéphane Venisse, Valerie Legué, Patricia Roeckel-Drevet

**Affiliations:** ^1^Clermont Université – Université Blaise Pascal, UMR 547 PIAFAubière, France; ^2^INRA, UMR 547 PIAFClermont-Ferrand, France

**Keywords:** amyloplast, gravisensing, membrane receptor, reaction wood, root growth, stem growth

## Abstract

Gravity is a crucial environmental factor regulating plant growth and development. Plants have the ability to sense a change in the direction of gravity, which leads to the re-orientation of their growth direction, so-called gravitropism. In general, plant stems grow upward (negative gravitropism), whereas roots grow downward (positive gravitropism). Models describing the gravitropic response following the tilting of plants are presented and highlight that gravitropic curvature involves both gravisensing and mechanosensing, thus allowing to revisit experimental data. We also discuss the challenge to set up experimental designs for discriminating between gravisensing and mechanosensing. We then present the cellular events and the molecular actors known to be specifically involved in gravity sensing.

## INTRODUCTION

Among the factors that influence the growth orientation in plants (e.g., light, gravity, water availability, and touch), gravity represents one of the most important environmental signals. This biological process known as gravitropism, starts from seed germination through the upright growth of shoots, ensuring the photosynthesis and the reproduction as well as the dispersion of seeds, and the downright growth of roots, supplying the plant in water and nutrients. When a plant organ is tilted, it adjusts its growth orientation relative to gravity direction, which is achieved by a curvature of the organ. Growth reorientation is the result of a differential cell elongation rate between the two sides of organs undergoing primary growth (Barlow and Rathfelder,1985; Tomos et al., 1989). In trees and perennial plants, the active cambium initiated in organs undergoing secondary growth contributes to the reorientation of the shoot through the differentiation and shrinkage of reaction wood (RW – i.e., tension wood or compression wood in eudicotyledonous and conifers respectively; IAWA, 1964; Archer, 1986). Gravitropism is therefore essential in the control of the posture and the form of land plants (Coutand et al., 2007; Moulia et al., 2011).

## MODELS DESCRIBE THE GRAVITROPIC MOVEMENTS IN PLANTS

Mathematic and kinematic tools have been extensively used for describing and quantifying the gravitropic movements in plants, and have been recently supplemented by integrative models. Even if gravity sensing events are not yet completely deciphered, these tools provide essential information to address the complex molecular and cellular mechanisms involved in gravitropism.

The time course of gravitropic curvature investigated in hypocotyl, stem, as well as in the trunk and branches illustrate the following steps in several species: the upward curving of the organs is observed after a latency phase and progressively followed by a “decurving” which starts at the tip and propagates downward. This latest has been described as autotropic (Firn and Digby, 1979) and may occur before the tip reaches the vertical (Firn and Digby, 1979; Stankovic et al., 1998).

Curvature time course in growing organs was initially calculated using the inclination angle of the organ’s tip relative to the vertical, and revealed that the gravitropic curvature obeys the so-called sine law (Sachs, 1882; review, Moulia and Fournier, 2009). The sine law represents the size of the gravitropic stimulus (S_gravi_) as equal to g sin γ, where g is the gravitational acceleration and γ the inclination angle. In other words this law predicts that the amplitude of gravitropism depends of the sinus of inclination angle. Even if this sine law has been confirmed in several species both in stems and roots, it is valid only in a limited range of inclination angles, ranging from 0 to 90° (see introduction in Göttig and Galland, 2014) and therefore, it does not characterize gravitropic movements of the whole organ over-time (reviewed in Moulia and Fournier, 2009). Later on, a curvature angle which is the change in tip inclination angle over time was proposed (Galland, 2002; Perbal et al., 2002; Hoshino et al., 2007). This parameter again is not satisfactory especially because it was not measured using the same reference from one experiment to another (horizontal, vertical, initial position of the tilted stem). Starting from the observation that the gravitropic responses of aerial organs showed general curving followed by basipetal straightening (Pickard, 1985), Bastien et al. (2013) proposed a model that takes into account the sensing of the local inclination angle but also of the local curvature, which progressively takes place. The sensing of the local inclination reflects the gravisensing mechanisms while the sensing of the local curvature could be referred as mechanosensing which has been described as graviproprioceptive. The authors defined a measurable ratio B that is a ratio between graviceptive and proprioceptive sensitivities. B was shown to control crucial aspects of the dynamics of the gravitropic response. The curving and decurving phases initially described as sequential, are in fact concomitant and linked to the initial degrees of inclination and curvature (Bastien et al., 2013). Recently, Bastien et al. (2014) extended this model by taking into account the growth effects, considered as the motor of movement, i.e., expansion of the curved zone and immobilization of the curvature state at elongation zone boundary (Selker and Sievers, 1987; Ishikawa and Evans, 1993). This model highlights that stems in primary growth rapidly straightened as to escape the growth destabilizing effects. To our mind, these findings precised the notion of autotropism as a reorientation of the axis controlled by internal cues such as the organ curvature. The consequence of the autotropic decurving is that RW and/or increased cell elongation occur alternatively from one side of the stem to the other.

Despite the fact that proprioceptive sensitivity has not been integrated in models of root gravitropism, it seems that the autotropic decurving has been observed during the last step of the gravitropic response in stems and as well in roots. Curiously, the analysis of the position of the lentil root tip and the root curvature as a function of time in microgravity revealed that the embryonic root curved strongly away from the cotyledons and then straightened out slowly following hydration (Perbal and Driss-Ecole, 2003; Driss-Ecole et al., 2008), suggesting an autotropic decurving in the absence of gravity signal. It is not clear if this decurving could occur in soil which structure can sometimes greatly restrict root growth and where the root system is mediated by a wide variety of processes including nutrient and water uptake, anchoring and mechanical support.

Another parameter that has been explored for elucidating the gravisensing mechanisms is the measurement of thresholds. Detailed kinetics of gravitropic curvature in horizontally stimulated roots have been reported in several studies and revealed for example that maize roots oriented at <40° from the vertical, overshot the vertical and then oscillated around this axis (Barlow et al., 1993). The angle of 10° seemed to be the minimum angle to induce a gravitropic response. On the contrary, when roots were tilted at more than 60°, verticality was hardly achieved. It is interesting to note that comparable thresholds occurred in root and stem. When coleoptiles were tilted at an angle of <10° from the vertical, the gravitropic response did not happen (Iiono et al., 1996). The first models of differential root growth leading to curvature took into account the presentation time (minimal duration of stimulation in the gravitational field; Larsen, 1957), in which the response was the function of the logarithm of the stimulus. Later, Perbal et al. (2002) observed that the hyperbolic model (H), related to a ligand-receptor system response, fitted better the experimental data. Other models took into account the differential growth among opposite cell lineages (Zieschang et al., 1997). Another interesting parameter used for approaching gravisensing mechanisms is the estimation of threshold acceleration perceived by organs. Lentil seedlings were grown in microgravity and subjected to low accelerations for several hours (Driss-Ecole et al., 2008). In these conditions, threshold acceleration perceived was inferior to 2.0 × 10^-3^*g*.

## MOST EXPERIMENTAL DESIGNS DO NOT ALLOW TO DISCRIMINATE BETWEEN GRAVISENSING AND MECHANOSENSING

As demonstrated above through mathematical models of stem gravitropic movements (Bastien et al., 2013, 2014), both gravisensing and mechanosensing lead to the reorientation of the plant. It is not clear whether gravisensing and mechanosensing act through the same mechanisms, and to what extent one can differentiate these stimuli. Trewavas and Knight (1994) considered that gravisensing is derived from an ancestral touch perception apparatus.

Mechanosensing occurs when plants are touched. Jaffe (1973) used the term of thigmomorphogenesis when describing the growth response of plants over time following repeated touching. In the literature numerous studies referring to mechanical stimulation concerned the response induced by external loading (Chehab et al., 2008) demonstrating that mechanical cues from the environment are sensed by the plant. Mechanical stresses are also intrinsic to plants and an increasing number of studies illustrate the occurrence of mechanosensing in cells and organs and its importance for the shape determination (Mirabet et al., 2011; Hamant, 2013). For example, it has been demonstrated that cells in *Arabidopsis* shoot apical meristem respond to local mechanical stresses by reorienting their growth, thereby guiding morphogenesis (Uyttewaal et al., 2012).

A gravistimulation as such should induce neither organ deformation nor touch. In several gravitropism studies, the plant or the organ have been tilted without being staked before (Azri et al., 2009; Tocquard et al., 2014b). Although such conditions allowed gravitropic movements, they also allowed organ bending under its own weight. This deformation of the organ can be considered as a thigmomorphogenetic stimulus (Coutand, 2010). In this context, both mechanosensing and gravisensing occur. Alternatively, staking of plants just before tilting might induce touch gene expression that could also interfere with graviresponse pathways. It remains a challenge to find an experimental design, which could allow discriminating between gravi and mechanosensing mechanisms.

## IDENTIFICATION OF CELLULAR AND MOLECULAR ACTORS IN GRAVISENSING MECHANISMS

### THE GRAVI-SENSING SITES

The most challenging research question is the identification of the tissues and/or cells able to sense and then perceive changes in the gravity vector.

**FIGURE 1:**
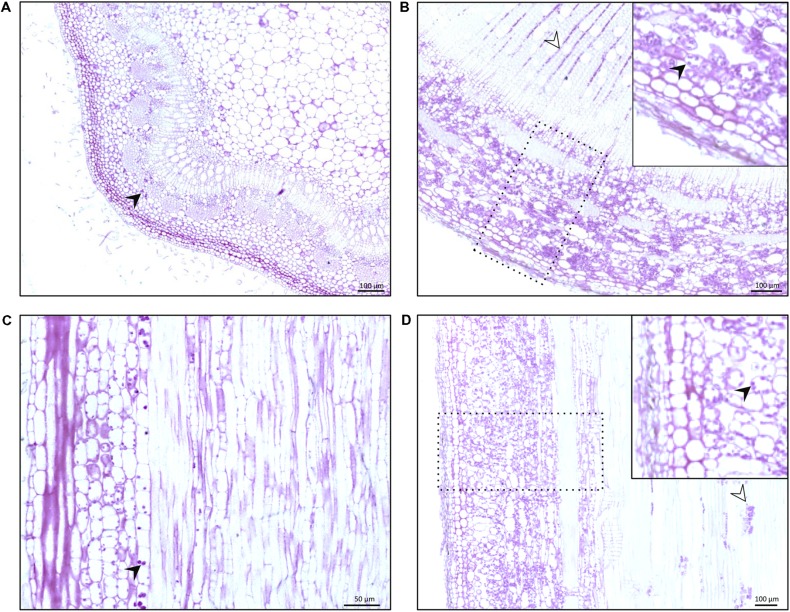
**Transversal **(A,B)** and longitudinal **(C,D)** stem sections of poplar tree, *Populus tremula* × *alba*, stained with Periodic Acid-Schiff (PAS).** This stain reveals the presence of starch and polysaccharides in dark purple. **(A,C)** Arrows indicate the presence of starch rich-amyloplasts in the endoderm of the primary growth-stem, at 3 cm from the stem apex. **(B,D)** Arrows and rectangles indicate wood rays and bark of secondary growth stem, respectively. Both tissues contain starch rich-granules.

Much insight on plant response to gravity is obtained by the study of organs exhibiting primary growth. The root columella located inside the root cap, which comprises polarized cells, is considered to be the key site of gravity sensing and perception. Columella cells contain starch-filled amyloplasts able to move under a change of gravity direction. The singularity of these organs is the spatial separation of the perception site from the responsive zone. Conversely, gravity sensing and response occur in the same region of young stems. The endoderm, located between the epiderm and the phloem, is considered as the gravi-sensing site. This tissue contains amyloplasts in young stems of herbaceous and ligneous species such as poplar (**Figures 1A,C**; Azri et al., 2013).

What happens in organs showing secondary growth? It is not possible to identify the endoderm in tree shoots since most bark cells are filled with starch (**Figures 1B,D**). Hence, the gravisensing cells are not identified yet neither are the gravisensing mechanisms (Tocquard et al., 2014b) leading to RW formation. RW can be induced by inclining a staked tree (Coutand et al., 2014) which suggests the modulation of cambial activity by gravistimulation *per se*, that occurs without the influence of mechanical deformation of the stem. Even if the gravisensing site for root undergoing a secondary growth in root is not yet identified, one could question if the cambium could be considered as an additional gravi-sensing site in roots.

### PROPOSED CONCEPTS AND MOLECULAR ACTORS

Despite, or maybe because of, the lack of indisputable protocol for the study of gravisensing in plants, various (opposite or complementary?) concepts are proposed related to the perception-transduction of the gravitropic stimulus. The starch-statolith hypothesis (Sack, 1997) explains that the direction of gravity is perceived by the plant through the sedimentation of starch-filled amyloplasts, named statolith, within specialized cells. The gravitational pressure hypothesis (Staves, 1997) suggests that mechanical deformation of the protoplast, cytoskeleton and cell wall components is the starting event of gravitropism. Another concept called “the tensegrity concept” (Ingber, 1997) assumes that the membrane is outstretched on the cytoskeleton backbone and that this system is in a state of equilibrium, between tensile and compressive forces. This concept is very suitable for explaining the perception of mechanical stress at the cell surface and the transmission to the intracellular compartment. The common idea that gravity-induced effects are initiated within the cells (Trewavas and Knight, 1994), is compatible with the tensegrity model which proposes that gravistimulation may unbalance the tensegrity forces and trigger cellular responses via membrane kinase proteins (Volkmann and Baluška, 2006). A complementary concept proposes that plant cells could sense gravity using the cytoskeleton-plasma membrane-cell wall continuum (CPMCW; Pickard and Ding, 1993; Baluška et al., 2003). Baluška and Volkmann (2011) discussed these different theories. Whichever theory is applied, the role of the amyloplasts and of the CPMCW with possible protein linkers seems realistic.

In the literature, some arguments corroborate parts of these hypothetic mechanisms. In roots, it has been suggested that the gravity-induced movements of amyloplasts could activate mechanosensing ion channels either in the amyloplast envelope or in reticulum endoplasmic and/or plasma membrane (Boonsirichai et al., 2002; Perbal and Driss-Ecole, 2003). These mechanosensing channels could be considered as gravi-receptors in inducing calcium dependent-signaling pathways. More recently it has been shown that plastids participate to root gravitropism not only through their sedimentation but also they likely play a role in the signal transduction pathway through the Translocon of the Outer envelope of the Chloroplast (TOC; Strohm et al., 2014). These findings implicate the functional interaction between plastids and actin cytoskeleton possibly via functions of TOC. In the same way, the investigation of plastid behavior in stem clearly demonstrates their role in gravi-perception (Morita and Nakamura, 2012). The plastid movements in stem are affected by the large and central vacuole of the endodermal cells. Moreover, genetic screening for *Arabidopsis* mutants with modified shoot gravitropism indicated that the vacuole is important for gravity perception (Morita, 2010).

More generally, within a putative perceptive cell, several molecular candidates could play a role in gravisensing. Two types of receptors could be involved including mechanosensitive ion channels and receptor like kinases (RLK). RLK are transmembrane proteins, composed of one or more extracellular domains, a single transmembrane domain and an intracellular kinase domain (Lehti-Shiu et al., 2009; Gish and Clark, 2011). RLK could act as sensors of the cell wall and restore its status to the cell wall by phosphorylation of the kinase domain. Among RLK, wall associated kinase (WAK) and *Catharanthus roseus* RLK1-like subfamilies are proposed to be cell wall status sensors (Gish and Clark, 2011; Engelsdorf and Hamann, 2014; Tocquard et al., 2014a). They could be involved in gravisensing by perceiving the deformation between the cell wall and the plasma membrane. Gens et al. (2000) hypothesized the existence of an architectural organization involving WAK, arabinogalactan proteins (AGP) at the interface between cytoplasm and cell wall. Several studies also showed the upregulation of AGP in response to gravistimulation (Lafarguette et al., 2004; Azri et al., 2014). This “plasmalemmal reticulum” could play a critical role in mechanosensing and possibly gravisensing (Gens et al., 2000). More recently, it has been shown that mechanosensitive channels including MCA2 could be also involved in gravisensing (Monshausen and Haswell, 2013; Iida et al., 2014).

The plant cytoskeleton is considered as a major receiver as well as transducer of mechanical signals. Nick (2011) presented the cytoskeleton as a tensegrity sensor. In this model, microfilaments (MF) are considered as the contractile and tensile elements while the microtubules (MT) are more rigid and resistant to compression. Bancaflor (2013) highlighted the apparent inconsistencies about the effects of actin inhibitory compounds on root gravitropism, and proposed models for how MF might regulate negatively gravitropism. The full understanding of the MF involvement in gravitropism has also to take into account the differences in actin organization between the root columella and the shoot endodermis cells, the former having first fine and short MF while the latter contain distinct F-actin bundles (Morita, 2010; Bancaflor, 2013). Several authors have suggested that gravitropic bending can trigger altered MT organization (Ikushima and Shimmen, 2005; Jacques et al., 2013; Toyota and Gilroy, 2013). In addition, gravitropism can be inhibited by antimicrotubular drugs or mutations affecting the dynamics of MT (Nick, 2012). On the contrary, tropic bending occurred in roots pretreated with microtubule depolymerizing agents (Bisgrove, 2008). These observations and others (Bisgrove, 2008) do not allow to discriminate the involvement of MT in gravisensing versus graviresponse, i.e., gravitropic bending. The difficulty to univocally show that the cytoskeleton is a tensegrity sensor may come from the fact that most studies examined either the involvement of MF or MT (Nick, 2013; Tatsumi et al., 2014) in gravitropism, although the cytoskeleton is far more complex. Indeed, evidence was brought that functional and structural interactions occurred between MT and actin, and that numerous proteins interacted with the cytoskeleton (Collings et al., 1998; Kotzer and Wasteneys, 2006).

## HOW TO GO FURTHER TO GRASP GRAVISENSING?

As just highlighted above, it is crucial to reliably discriminate gravisensing from mechanosensing, and the same goes for sensing from the signal transduction and early responsive elements. According to Nick (2011) clear concepts of the sensing mechanisms have to be elaborated in order to design unequivocal experimental approaches. Incidentally, the effect of the direction of light as well as the light quality have also to be taken into account in designing an experiment that wish to focus on gravisensing and graviresponse as multiple light signaling pathways interact with gravitropism (Mullen and Kiss, 2008). Importantly, to our mind the gravistimulation should not cause bending that could lead to organ and tissue deformation. Consequently, the study of gravisensing has to be done before any curving response occurred. Another way is the utilization of microgravity conditions through space experiments (Ruyters and Braun, 2014).

Furthermore, there is a general consensus on the identity of the gravisensing cells in primary shoot and root while these cells remained to be localized in organs driven by secondary growth. Further insights on this subject are impaired by the compulsory use of ligneous species models. For instance, in the ligneous model *Populus trichocarpa*, very few mutants are available compared to *Arabidopsis thaliana*, which was used in most studies. One can even ask if the tissues in secondary growth are able to perceive gravity or if they respond to a signal coming from the apexes.

Another challenge is to identify the gravity receptors in roots and in stems. Approaches such as transcriptomics and proteomics combined with the study of mutants could lead to the inference of a network of genes involved in gravisensing. In addition, it will be interesting to investigate the functional interaction between the cytoskeleton and gravi-sensors. In parallel, modelization of the mechanical deformation of the cytoskeleton could help to understand the function of the cytoskeleton network in gravitropism.

## Conflict of Interest Statement

The authors declare that the research was conducted in the absence of any commercial or financial relationships that could be construed as a potential conflict of interest.
